# The effect of using a robust optimality criterion in model based adaptive optimization

**DOI:** 10.1007/s10928-017-9521-5

**Published:** 2017-04-06

**Authors:** Eric A. Strömberg, Andrew C. Hooker

**Affiliations:** Pharmacometrics Research Group, Department of Pharmaceutical Biosciences, Uppsala University, Uppsala, Sweden

**Keywords:** Model based adaptive optimal design, Dose optimization, Robust optimal design, ELD, API, Study design

## Abstract

Optimizing designs using robust (global) optimality criteria has been shown to be a more flexible approach compared to using local optimality criteria. Additionally, model based adaptive optimal design (MBAOD) may be less sensitive to misspecification in the prior information available at the design stage. In this work, we investigate the influence of using a local (lnD) or a robust (ELD) optimality criterion for a MBAOD of a simulated dose optimization study, for rich and sparse sampling schedules. A stopping criterion for accurate effect prediction is constructed to determine the endpoint of the MBAOD by minimizing the expected uncertainty in the effect response of the typical individual. 50 iterations of the MBAODs were run using the MBAOD R-package, with the concentration from a one-compartment first-order absorption pharmacokinetic model driving the population effect response in a sigmoidal EMAX pharmacodynamics model. The initial cohort consisted of eight individuals in two groups and each additional cohort added two individuals receiving a dose optimized as a discrete covariate. The MBAOD designs using lnD and ELD optimality with misspecified initial model parameters were compared by evaluating the efficiency relative to an lnD-optimal design based on the true parameter values. For the explored example model, the MBAOD using ELD-optimal designs converged quicker to the theoretically optimal lnD-optimal design based on the true parameters for both sampling schedules. Thus, using a robust optimality criterion in MBAODs could reduce the number of adaptations required and improve the practicality of adaptive trials using optimal design.

## Introduction

When designing a future study, what is thought to be known beforehand might not be a good representation of the truth. Whether deciding upon sample size, sampling schedule or which covariate to prioritize, the traditional experimental design approaches are dependent on the prior information and the resulting design will be based on an expectation of reality. With the rising use of nonlinear mixed-effects models (NLMEM) in drug development in the past decades, new tools have become available to predict what to expect in more intricate detail by study simulation. As an extension of the NLMEM, model based optimal experimental design (OD) has become a recognized methodology for the design of pharmaceutical studies. In OD, the informativeness of a potential design is summarized in the Fisher information matrix (FIM) [1]. By finding the design which maximizes FIM, the expected variance–covariance matrix can be minimized according to the Cramer–Rao inequality [2, 3].

Several optimality criteria have been developed to allow for numerical comparison of FIMs when optimizing the designs. The most common criteria are D-optimality, in which the determinant of the FIM is maximized to find the best design, and lnD-optimality where the natural logarithm of the determinant of the FIM is maximized. D-optimality and lnD-optimality are considered local criteria which assume that model parameters are known single point values. There are however criteria for global (robust) design in which only prior distributions of the parameters are assumed to be known. Global design criteria have been shown to be more flexible than local criteria and may be less sensitive to parameter misspecification in the design stage [1, 4–7]. One example of a global design criterion is ELD-optimality where the expectation of the natural logarithm of the determinant of the FIM for the prior distribution of parameters is maximized (the criterion is also known as API optimality [8]). Regardless of the chosen optimality criterion, a design optimized using the regular model based OD methodology will be dependent on the prior information regarding the model and parameters. If the prior is not a good representation of the truth, the design will be based on a misspecified guess of the model and parameters, potentially resulting in a sub-optimal design. Thus even when using OD for the design of a future study, there is a risk of not finding a desired outcome due to misspecified prior information in the design stage.

However, adaptive designs approaches for sequential trials such as model based adaptive optimal design (MBAOD) have been shown to be less sensitive to misspecified prior information in the design stage [9, 10]. Instead of preforming the optimal design for the entire study population based on the information available prior to the trial, as in a regular OD, the MBAOD approach divides the study population into smaller cohorts. At the initial cohort, the design is based on the prior information. However, for the following cohorts, the prior information regarding the parameters and model can be updated with the information gained from the previous cohort. Thus, the adaptive design approach will continuously improve the parameter and model guess for the subsequent cohorts. This iterative approach of guessing, learning, and improving the design, may continue until an entire study population has been enrolled. Additionally, the MBAOD can be stopped before recruitment of all available subjects using a stopping criterion for the MBAOD. The stopping criterion, which can be based on, for example, a maximum parameter variability or accuracy of the model prediction, can then be evaluated at the end of each cohort to determine if the study should be stopped.

The aim of this work is to investigate any potential advantage in using robust design criteria in MBAODs of a simulated pharmacokinetic–pharmacodynamics (PKPD) study when the dose of a hypothetical drug is optimized.

For the purpose of the study simulation, the prior information regarding pharmacodynamics (PD) is assumed to be misspecified, resulting in a 50% over estimated initial guess of the PD fixed effect parameters. The pharmacokinetics (PK) of the PKPD model is assumed to be well characterized with fixed parameter values to reduce the complexity of the parameter estimation. No model misspecification is included. The stopping criteria for the MBAOD assumes that the PD fixed effect curve will have a 95% confidence interval (CI) within 60–140% of the population mean effect prediction, for all sampling points and included doses.

For a rich and sparse sampling schedule, and using lnD and ELD optimality criterion, the design efficiency of the chosen doses for each cohort in the MBAODs is compared relative to an lnD-optimal design based on the true parameters. Additionally, the potential influence of the optimality criterion on the number of individuals required to reach the stopping criteria of the MBAOD, final parameter relative estimation error and the overall dose selection is evaluated.

## Theoretical

With the vector of fixed effect parameters $\beta = \left[ {\beta_{1} , \ldots ,\beta_{j} } \right]$ and the vector of random inter-individual deviations from the fixed effects $\eta_{i} = [\eta_{i,1} , \ldots , \eta_{i,k} ]$ the *i*th individual’s parameter vector $\theta_{i}$ is described by $\theta_{i} = g(\beta , \eta_{i} )$. The *i*th individual’s response $y_{i}$ can be given by the response function *f* and the residual error function *h,* describing the random unexplained variability (RUV), as

1 $$y_{i} = {\text{f}}\left( {\theta_{i} , \xi_{i} } \right) + {\text{h}}\left( {\theta_{i} , \xi_{i} , \varepsilon_{i} } \right)$$

where $\xi_{i}$ is the individual design vector containing design variables such as sampling times and covariates and $\varepsilon_{i}$ is the residual error vector. For simplicity, with no covariance in the random effects, $\eta_{i}$ and $\varepsilon_{i}$ are sampled normal distributions with mean 0 and covariance matrix $\varOmega = {\text{diag}}(\omega_{1}^{2} , \ldots ,\omega_{k}^{2} )$ and $\sum = {\text{diag}}(\sigma_{1}^{2} , \ldots ,\sigma_{m}^{2} )$ respectively. The vector of random effects can thus be constructed as $\lambda = [\omega_{1}^{2} , \ldots ,\omega_{k}^{2} ,\sigma_{1}^{2} , \ldots ,\sigma_{m}^{2} ]$ giving the vector of population parameters $\varTheta = \left[ {\beta ,\lambda } \right] = \left[ {\beta_{1} , \ldots ,\beta_{j} ,\omega_{1}^{2} , \ldots ,\omega_{k}^{2} ,\sigma_{1}^{2} , \ldots ,\sigma_{m}^{2} } \right]$. The expected model response ${\text{E}}\left( {y_{i} } \right)$ and variance ${\mathbf{V}}(y_{i} )$ often must be approximated since $f\left( {\theta_{i} , \xi_{i} } \right)$ may lack an exact solution due to non-linearity with respect to the random effect parameters. Therefore, the model in Eq. (1) is linearized, often using a first order Taylor expansion, with respect to *η*_*i*_ and *ε*_*i*_ to guarantee marginal normally distributed observations. The fastest and simplest approximation of the model is the first order approximation (FO) where the model is linearized around the typical values *η*_*i*_ = 0 and *ε*_*i*_ = 0 [11].

If the expectation of response ${\text{E}}\left( {y_{i} } \right)$ and variance ${\mathbf{V}}(y_{i} )$ are normally distributed, the subject specific fisher information matrix **FIM**_i_ can be written as [8]

2 $${\mathbf{FIM}}_{i} (\varTheta ,\xi_{i} ) = \frac{1}{2}\left[ {\begin{array}{*{20}c} {\varvec{A}({\text{E}}(y_{i} ),{\mathbf{V}}(y_{i} ))} & {\varvec{C}({\mathbf{V}}(y_{i} ))} \\ {\varvec{C}({\mathbf{V}}(y_{i} ))} & {\varvec{B}({\mathbf{V}}(y_{i} ))} \\ \end{array} } \right]$$

where

$$\varvec{A}\left( {{\text{E}}(y_{i} ),{\mathbf{V}}(y_{i} )} \right) = 2 \cdot \frac{{\partial {\text{E}}\left( {y_{i} } \right)}}{\partial \beta }^{T} {\mathbf{V}}\left( {y_{i} } \right)^{ - 1} \cdot \frac{{\partial {\text{E}}\left( {y_{i} } \right)}}{\partial \beta } + \,\,tr\left( {\frac{{\partial {\mathbf{V}}\left( {y_{i} } \right)}}{\partial \beta } \cdot {\mathbf{V}}\left( {y_{i} } \right)^{ - 1} \cdot \frac{{\partial {\mathbf{V}}\left( {y_{i} } \right)}}{\partial \beta } \cdot {\mathbf{V}}\left( {y_{i} } \right)^{ - 1} } \right)$$

$$\varvec{B}\left( {{\mathbf{V}}(y_{i} )} \right) = tr\left( {\frac{{\partial {\mathbf{V}}\left( {y_{i} } \right)}}{\partial \lambda } \cdot {\mathbf{V}}\left( {y_{i} } \right)^{ - 1} \cdot \frac{{\partial {\mathbf{V}}\left( {y_{i} } \right)}}{\partial \lambda } \cdot {\mathbf{V}}\left( {y_{i} } \right)^{ - 1} } \right)$$

$$\varvec{C}\left( {{\mathbf{V}}(y_{i} )} \right) = tr\left( {\frac{{\partial {\mathbf{V}}\left( {y_{i} } \right)}}{\partial \beta } \cdot {\mathbf{V}}\left( {y_{i} } \right)^{ - 1} \cdot \frac{{\partial {\mathbf{V}}\left( {y_{i} } \right)}}{\partial \beta } \cdot {\mathbf{V}}\left( {y_{i} } \right)^{ - 1} } \right)$$

Furthermore, ${\mathbf{FIM}}_{i}^{ }$, can be reduced to its block-diagonal form if the fixed effects are assumed to be independent on the random effects accordingly [12]

3 $${\mathbf{FIM}}_{i}^{block - diag} (\varTheta ,\xi_{i} ) = \frac{1}{2}\left[ {\begin{array}{*{20}c} {\varvec{A}({\text{E}}\left( {y_{i} } \right),{\mathbf{V}}\left( {y_{i} } \right))} & {\mathbf{0}} \\ {\mathbf{0}} & {\varvec{B}({\text{E}}\left( {y_{i} } \right),{\mathbf{V}}\left( {y_{i} } \right))} \\ \end{array} } \right]$$

with

$$\varvec{A}\left( {{\text{E}}(y_{i} ),{\mathbf{V}}(y_{i} )} \right) = 2 \cdot \frac{{\partial {\text{E}}\left( {y_{i} } \right)}}{{\partial \beta_{ } }}^{T} {\mathbf{V}}\left( {y_{i} } \right)^{ - 1} \cdot \frac{{\partial {\text{E}}\left( {y_{i} } \right)}}{{\partial \beta_{ } }}$$

If the study consists of N independent groups with *s*_i_ individuals per group, and all individuals in a group have the same elementary design then the population FIM can be constructed as the sum of FIMs for all groups as

4 $${\mathbf{FIM}}\left( {\varTheta ,\,\xi } \right) = \mathop \sum \limits_{i = 1}^{N} s_{i} \cdot {\mathbf{FIM}}_{i} \left( {\varTheta ,\,\xi_{i} } \right) .$$

where $\xi = \left[ {\xi_{1} , \ldots ,\xi_{i} } \right]$ contains all elementary designs.

The design criteria compared in this work are lnD-optimality and ELD-optimality which finds the design that maximizes the block-diagonal **FIM** accordingly

5 $$\xi_{lnD} = \mathop{{\text{arg max}}}\limits_{\xi}\, \ln \left( {\left| { {\mathbf{FIM}}^{block - diag} \left( {\varTheta ,\xi } \right)} \right|} \right)  $$

and

6 $$\xi_{ELD} = \mathop{{\text{arg max}}}\limits_{\xi}\, E_{\varTheta } \left[ {{ \ln }\left( {\left| { {\mathbf{FIM}}^{block - diag} \left( {\varTheta ,\xi } \right)} \right|} \right)} \right]$$

where $E_{\varTheta }$ is the expectation over the prior distribution of the parameters $\varTheta$. For a more comprehensive description on the derivation of the FIM, for different linearizations, and additional design criteria see [8, 11, 12].

## Methods

In this work, the response model *f* was described by a sigmoidal E_max_ PD model being driven by drug concentration described by a one-compartment first order absorption PK. The PK model was assumed to be known and fixed with parameter values based on the warfarin PK model used in [13, 14]. The RUV of the PD model was given by a combined additive and proportional residual error model. The *i*th individual’s effect response *y*_*i*_ was given by

7 $$y_{i} \left( {C_{i} ,\theta_{i} } \right) = \left( {\beta_{Base} + \frac{{C_{i}^{{\beta_{\gamma } }} \cdot E_{\hbox{max} ,i} }}{{C_{i}^{{\beta_{\gamma } }} + EC50_{i}^{{\beta_{\gamma } }} }}} \right) \cdot \left( {1 + \varepsilon_{prop,i} } \right) + \varepsilon_{add,i}$$

8 $$C_{i} \left( {t_{i} ,\theta_{i} } \right) = \frac{{Dose \cdot \beta_{Ka} }}{{ {V_{i} \cdot {\beta_{Ka} - {Cl_{i} }} }}}\cdot \left( {e^{{ - \frac{{Cl_{i} }}{{V_{i} }} t_{i} }} - e^{{ - \beta_{Ka} t_{i} }} } \right) \;({\text{mg}}/{\text{L}})$$

9 $$EMAX_{i} = \beta_{Emax} \cdot e^{{\eta_{Emax,i} }}$$

10 $$EC50_{i} = \beta_{EC50} \cdot e^{{\eta_{EC50,i} }} \;({\text{mg}}/{\text{L}})$$

11 $$Cl_{i} = \beta_{CL} \cdot e^{{\eta_{CL,i} }} \;({\text{L}}/{\text{h}})$$

12 $$V_{i} = \beta_{V} \cdot e^{{\eta_{V,i} }} \;(L)$$

where *t*_*i*_ is the individual vector of time points at which the system response is evaluated.

## Design setup

Using the response model described above, a MBAOD for a dose optimization trial was run 50 times using the MBAOD R-package [15] in four possible design scenarios; lnD or ELD-optimality for a sparse sampling schedule with three samples at t_sparse_ = (0.5,3,60) h, and lnD or ELD-optimality for a rich sampling schedule with eight samples t_rich_ = (0.5, 2, 3, 6, 24, 36, 72, 120) h. The sampling schedules were fixed and the same for all individuals and groups. For the optimizations using ELD optimality, a normal distribution was assumed for the fixed effects parameters with mean $\beta_{j}$ and standard deviation $SD_j$. In the first cohort $SD_j$ was calculated from a 10% coefficient of variation and for the subsequent cohorts, $SD_j$ was updated from the parameter variance in the **COV**_β_ from the estimation using all previous cohorts. The expectation of the FIM was based on 10 samples from the parameter prior distribution using Latin hypercube sampling.

In all MBAODs the initial parameter guess was a +50% misspecification of the true fixed-effect model parameters (Table 1) and the dose was treated as a discrete covariate with the allowed range of 0–500 mg in integer steps. The initial design (before optimization) for the first cohort was two groups dosed with 0 and 160 mg of the hypothetical compound and four individuals per group. Subsequent cohorts added one additional group with two individuals receiving an optimized dose. A maximum of 50 cohorts was allowed. At the start of each cohort, the dose was optimized using the current, updated, parameter guesses (and for ELD-optimization the parameter uncertainties). Optimization was performed using the R-version of PopED [8, 16]. Following the design optimization, individual data was simulated using this design using the true parameter values. For all cohorts, the parameters were then estimated for all simulated data (including data from any previous cohorts) using the FOCEI algorithm in NONMEM 7.3 [17] via PsN version 4.5.16 [18]. After the estimation step the MBAOD entered the stopping criterion evaluation (described below) to determine whether a new cohort of (simulated) patients should be included in the trial. If the stopping criterion was not achieved, the MBAOD continued by updating the current guess of the parameters based on the information from all the previous cohorts and entered another cycle of design optimization, study simulation and parameter estimation.

**Table 1:** Parameter values of the PKPD response model for the true values used for simulation and the misspecified initial guess of the parameters (in bold in the far right column)

Parameter	Description	True	Guess
$\beta_{CL}$ (L/h)	Clearance	0.15 FIX	Same
$\beta_{V}$ (L)	Volume of distribution	8 FIX	Same
$\beta_{Ka}$ (mg/h)	Absorption rate	1 FIX	Same
$\beta_{Base}$ (–)	Baseline effect	1	**1.5**
$\beta_{EMAX}$ (–)	Maximum effect	100	**150**
$\beta_{EC50}$ (mg/L)	50% of maximum effective concentration	7	**10.5**
$\beta_{\gamma }$ (–)	Sigmoidicity Coefficient	2	**3**
$\omega_{ CL}^{2}$ (L/h)	Between subject variability of CL	0.07 FIX	Same
$\omega_{ V}^{2}$ (L)	Between subject variability of V	0.02 FIX	Same
$\omega_{ EMAX}^{2}$ (L/h)	Between subject variability of EMAX	0.0625	Same
$\omega_{ EC50}^{2}$ (L)	Between subject variability of EC50	0.0625	Same
$\sigma_{ add}^{2}$ (mg/L)	Additive residual error component	0.001 FIX	Same
$\sigma_{ prop}^{2}$ (–)	Proportional residual error component	0.015	Same

“FIX” indicates that the parameters were not estimated, but rather assumed known in both design optimization and parameter estimation

## Stopping criterion

In the stopping criterion, 100,000 fixed effect parameter vectors were generated with a multivariate student’s t-distribution (*rmvt*) from the *mvtnorm* R-package:

13 $$_{sim} \beta = rmvt(df,\widehat{\beta}, S)$$

where $_{sim} \beta$ is a matrix of 100,000 simulated parameter vectors, $\widehat{\beta }$ is a vector of the estimated fixed effect parameters from NONMEM, *df* is the degrees of freedom and S is the scale matrix. The degrees of freedom was dependent on the cumulative number of included individuals $n_{ID}$ and the number of estimated parameters accordingly:

14 $$df = n_{ID} - \left( {n_{{\hat{\beta }}} + \frac{{n_{{\hat{\lambda }}} }}{2}} \right)$$

where $n_{{\hat{\beta }}}$ and $n_{{\hat{\lambda }}}$ are the number of estimated fixed effect and random effect parameters respectively. Given the variance–covariance matrix for the fixed effects $COV_{{\hat{\beta }}}$ from NONMEM and the degrees of freedom *df,* the scale matrix **S** was constructed as

15 $${\mathbf{S}} = \frac{{{\mathbf{COV}}_{{\hat{\beta }}} (df - 2)}}{df}$$

The parameters in $_{sim} \beta$ were used by the model to simulate 100,000 population mean responses at the times specified by the sampling schedule vector *t,* for all dose arms included in the study. From the simulated responses, 95% CIs of the predicted response at each sample time for all doses was constructed. If all CIs fell within 60–140% of the population mean response for all sampling times and doses, the study was stopped.

### Comparison of designs

The MBAODs using lnD-optimality and ELD-optimality for rich and sparse sampling schedules were compared via the total required number of simulated individuals to reach the pre-determined stopping criteria and the relative estimation error of the final parameter estimates. The relative estimation error of the final estimate of parameter *j* in iteration *x,* $\beta_{j,x}$, was calculated by

16 $$REE\left( {\beta_{j,x} } \right) = \frac{{\widehat{\beta }_{j,x} - \beta_{j} }}{{\beta_{j} }}$$

where $\widehat{\beta }_{j}$ is the estimate x of the true value $\beta_{j}$ of parameter j.

Further, each design was compared to the theoretically best design, generated by simulating an MBAOD using lnD-optimality and while assuming the correct model parameter values and with no parameter updating in the MBAOD process. This comparison was done, first, by computing the efficiency of each MBAOD design $\widehat{\xi }$, based on the initial parameter guess $\widehat{\varTheta }$, relative to the theoretically best design for each cohort:

$$Efficiency = \left( {\frac{{\left| {{\mathbf{FIM}}\left( {\varTheta ,\widehat{\xi }} \right)} \right|}}{{\left| {{\mathbf{FIM}}\left( {\varTheta ,\xi } \right)} \right|}}} \right)^{1/P}$$

where $\xi_{ }$ is the optimal design based on the true parameters in $\varTheta$ and *P* is the total number of estimated parameters. From the multiple MBAOD simulations, the median design efficiency and a 95% non-parametric confidence interval was constructed from the 2.5th and 97th percentiles of design efficiency for each cohort. Second, the distribution of selected doses across all iterations for all designs was summarized in a histogram and compared to the the corresponding theoretically best design.

## Results

**Fig. 1:**
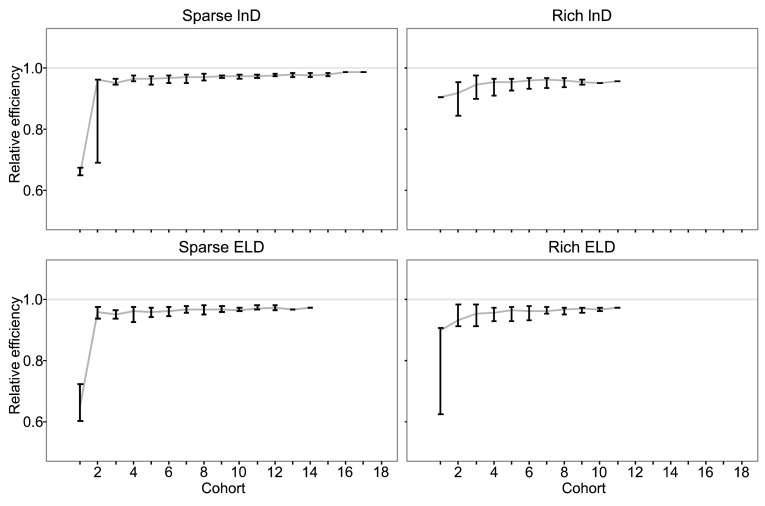
The efficiency of the MBAOD designs based on lnD (*Top*) and ELD (*Bottom*) optimality assuming 50% misspecification in PD fixed-effect parameters, relative to a lnD-optimal design based on the true parameter values, for the sparse (*Left*) and rich (*Right*) sampling schedules. The *line* and the *upper* and *lower bracket limits* represent the 50th, 2.5th and 97.5th percentiles of the achieved design efficiency after each adaptive cohort from 50 MBAOD simulations

With the initial parameter misspecification of +50% on all PD fixed effect parameters, the MBAODs using ELD-optimality had efficiencies that stabilized more quickly to values close to the theoretically optimal lnD-design based on the true parameters, for both sparse and rich sampling (Fig. 1). For the sparse sampling designs, the median result of the MBAODs using ELD and lnD had high efficiency even at the second cohort, but the variability in efficiency between MBAOD iterations was much smaller with the ELD designs compared to the lnD designs. After the second cohort of patients the ELD and lnD designs were very similar. For the rich sampling designs, the relative efficiencies of the designs were also high at the second cohort of patients. The efficiencies of the ELD designs between MBAOD iterations were somewhat higher than the lnD designs for the first four iterations, but from the fifth iteration and forward the designs were very similar.

**Fig. 2:**
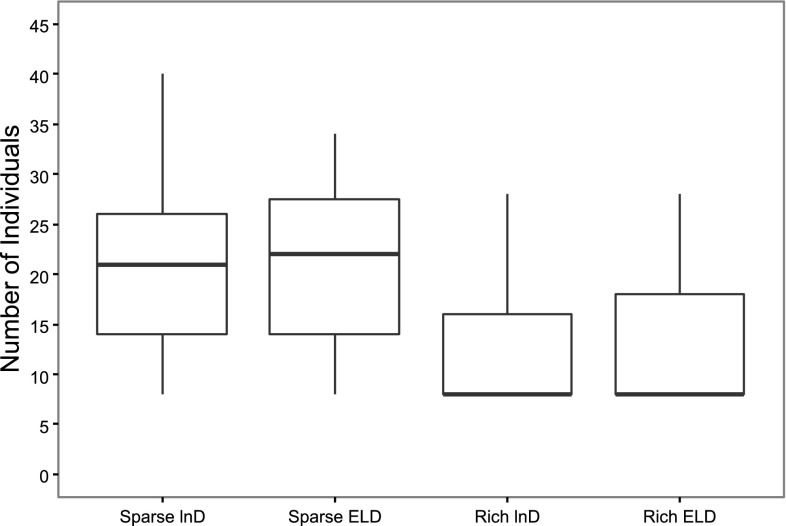
*Boxplots* of the total sample size required to reach the endpoint for 50 iterations of the model based adaptive optimal design using lnD-optimality and ELD optimality for sparse and rich sampling schedules

**Fig. 3:**
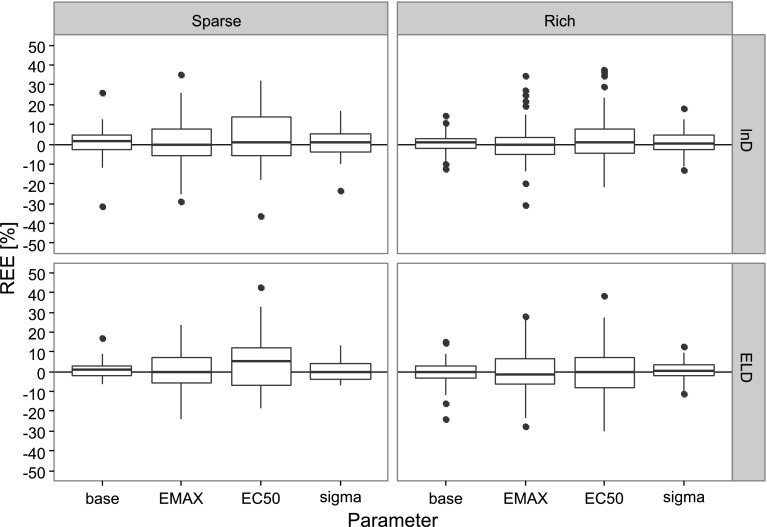
*Boxplots* of relative estimation error for the final parameter estimates in 50 iterations of the model based adaptive optimal design using lnD-optimality and ELD optimality for rich and sparse sampling schedules

**Fig. 4:**
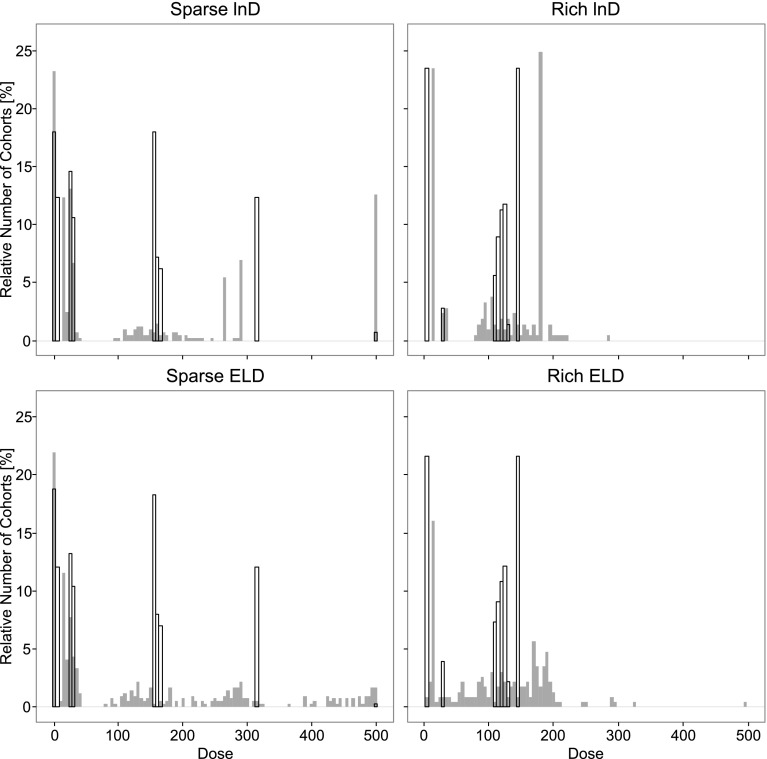
Histogram of the dose chosen (*Grey*) in all cohorts in 50 iterations of the model based adaptive optimal design using lnD-optimality(*Top*) and ELD optimality (*Bottom*) for sparse (*left*) and rich (*right*) sampling schedules. The *black outline* represents the dose selection by the theoretically best lnD optimal design based on the true parameter values for the same number of cohorts in each simulation

The number of individuals required to reach the stopping criteria of the MBAOD was similar between the lnD and ELD-optimal designs (Fig. 2). The maximum number of required cohorts and individuals was however less for the MBAOD using ELD-optimality in the sparse sampling schedule scenario (Figs. 1, 2). Additionally, there was little difference in the relative estimation error between the optimality criteria (Fig. 3). The ELD resulted in a more even distribution of chosen doses across all 50 iterations of the MBAOD and included the doses from the lnD-optimal design based on the true parameters more often than the MBAODs using lnD-optimality (Fig. 4).

## Discussion and conclusion

In this work, the effects of lnD and ELD optimality were compared for model based optimal designs of simulated dose optimization PKPD studies with rich and sparse sampling. A stopping criterion for the MBAOD was constructed based on the accuracy of the effect prediction. The designs from the two optimality criteria were compared in terms of required sample size, final parameters, dose selection and efficiency relative to the true lnD-optimal design.

There were differences in the MBAODs using lnD- and ELD-design calculations (Fig. 4) as well as in the speed at which the lnD or ELD design approached the efficiency of a lnD design calculated without model parameter misspecification. With the ELD designs achieving high efficiency after just 2–3 cohorts of patients, compared to 3–5 cohorts needed for the lnD designs. Additionally, design efficiencies in the second cohort were more variable when using lnD-optimality than ELD-optimality, indicating designs that are more sensitive to parameter estimates from the initial cohort of patients and less robust to parameter misspecification.

These differences were not noticeable in the number of individuals needed to achieve the MBAOD stopping criteria (Fig. 2), or in parameter bias (Fig. 3) for the 50 MBAOD iterations in this example. This apparent insensitivity to the lnD or ELD design criteria in the final parameter estimates and number of individuals in the study could clearly be influenced by many factors. First of all, only one set of true and misspecified parameters was investigated. The design setup (number of individuals per cohort, number of cohorts) and lowering the precision criteria (from 60 to 140% of the mean prediction to 80–120% for example) could also influence results. In addition, in this particular example, optimizing both dose and sample times could have shown more differences between the robust and local design criteria since it would require the optimizations to find more optimal design support points. Further, for the optimization using the ELD approach, only 10 samples were taken from the prior distribution due to long run times, a large variance of the ELD criterion may have destabilized the design. By increasing the number of samples, the ELD approach could potentially have been more robust.

However, in this example with these experimental settings we see that the adaptive properties of a MBAOD allow for local designs to be as robust as global optimality criteria given that the local designs are allowed to adapt a sufficient number of times. The MBAODs using ELD-optimality did however converge quicker towards the “true” design, resulting in a maximum number of required cohorts which was lower compared to the lnD–MBAOD. Additionally, the doses according to the theoretically true lnD-optimal design was included in the MBAOD more often by the robust design (Fig. 4). The main disadvantage of using ELD optimality in these examples was increased run-times for design optimization. However, compared to the time to execute the study on each adaptive cohort in a real practical setting, these additional run-times would be negligible. With the ELD MBAODs achieving more efficient and robust designs earlier in the adaptive process, this allows the MBAOD to approach a more practical 2-step or 3-step sequential design [19, 20]. Thus using a robust optimality criterion in MBAOD could be more practical for performing adaptive trials using optimal design.

## References

[1] Atkinson AC, Donev AN (1992). Optimum experimental design.

[2] Cramér H (1946) Methods of estimation. In: Mathematical methods of statistics. Princeton University Press. http://press.princeton.edu/titles/391.html. Accessed 3 Oct 2014

[3] Radhakrishna Rao C (1945). Information and accuracy attainable in the estimation of statistical parameters. Bull Calcutta Math Soc.

[4] Dokoumetzidis A, Aarons L (2007). Bayesian optimal designs for pharmacokinetic models: sensitivity to uncertainty. J Biopharm Stat.

[5] Tod M, Rocchisani J-M (1996). Implementation of OSPOP, an algorithm for the estimation of optimal sampling times in pharmacokinetics by the ED, EID and API criteria. Comput Methods Programs Biomed.

[6] Pronzato L, Walter E (1985). Robust experiment design via stochastic approximation. Math Biosci.

[7] Hooker AC, Foracchia M, Dodds MG, Vicini P (2003). An evaluation of population D-optimal designs via pharmacokinetic simulations. Ann Biomed Eng.

[8] Nyberg J, Ueckert S, Strömberg EA (2012). PopED: an extended, parallelized, nonlinear mixed effects models optimal design tool. Comput Methods Programs Biomed.

[9] Foo LK, Duffull S (2012). Adaptive optimal design for bridging studies with an application to population pharmacokinetic studies. Pharm Res.

[10] Maloney A, Karlsson MO, Simonsson USH (2007). Optimal adaptive design in clinical drug development: a simulation example. J Clin Pharmacol.

[11] Retout S, Mentré F (2003). Further developments of the Fisher information matrix in nonlinear mixed effects models with evaluation in population pharmacokinetics. J Biopharm Stat.

[12] Retout S, Duffull S, Mentré F (2001). Development and implementation of the population Fisher information matrix for the evaluation of population pharmacokinetic designs. Comput Methods Programs Biomed.

[13] Strömberg EA, Nyberg J, Hooker AC (2016). The effect of Fisher information matrix approximation methods in population optimal design calculations. J Pharmacokinet Pharmacodyn.

[14] Nyberg J, Bazzoli C, Ogungbenro K (2014). Methods and software tools for design evaluation for population pharmacokinetics-pharmacodynamics studies. Br J Clin Pharmacol.

[15] Hooker AC, van Hasselt C Platform for adaptive optimal design of nonlinear mixed effect models. In: PAGE 22 Abstr 2952. www.page-meeting.org/?abstract=2952

[16] Foracchia M, Hooker A, Vicini P, Ruggeri A (2004). POPED, a software for optimal experiment design in population kinetics. Comput Methods Programs Biomed.

[17] Beal S, Sheiner LB, Boeckmann A, Bauer RJ (2009). NONMEM user’s guides. (1989–2009).

[18] Lindbom L, Pihlgren P, Jonsson EN, Jonsson N (2005). PsN-Toolkit—a collection of computer intensive statistical methods for non-linear mixed effect modeling using NONMEM. Comput Methods Programs Biomed.

[19] Dette H, Bornkamp B, Bretz F (2013). On the efficiency of two-stage response-adaptive designs. Stat Med.

[20] Dragalin V, Fedorov VV, Wu Y (2008). Two-stage design for dose-finding that accounts for both efficacy and safety. Stat Med.

